# Rv2607 from *Mycobacterium tuberculosis* Is a Pyridoxine 5′-Phosphate Oxidase with Unusual Substrate Specificity

**DOI:** 10.1371/journal.pone.0027643

**Published:** 2011-11-14

**Authors:** Ellene H. Mashalidis, Tathagata Mukherjee, Paweł Śledź, Dijana Matak-Vinković, Helena Boshoff, Chris Abell, Clifton E. Barry

**Affiliations:** 1 National Institute of Allergy and Infectious Disease, National Institutes of Health, Bethesda, Maryland, United States of America; 2 Department of Chemistry, University of Cambridge, Cambridge, United Kingdom; University of Delhi, India

## Abstract

Despite intensive effort, the majority of the annotated *Mycobacterium tuberculosis* genome consists of genes encoding proteins of unknown or poorly understood function. For example, there are seven conserved hypothetical proteins annotated as homologs of pyridoxine 5′-phosphate oxidase (PNPOx), an enzyme that oxidizes pyridoxine 5′-phosphate (PNP) or pyridoxamine 5′-phosphate (PMP) to form pyridoxal 5′-phosphate (PLP). We have characterized the function of Rv2607 from *Mycobacterium tuberculosis* H37Rv and shown that it encodes a PNPOx that oxidizes PNP to PLP. The k_cat_ and K_M_ for this reaction were 0.01 s^−1^ and 360 µM, respectively. Unlike many PNPOx enzymes, Rv2607 does not recognize PMP as a substrate.

## Introduction

A large fraction of the *Mycobacterium tuberculosis* (*Mtb*) genome encodes proteins of unknown or putative function based on sequence homology to other characterized proteins [1]. This knowledge gap poses a significant challenge to efforts to understand the complete biological repertoire of this deadly pathogen. While bioinformatics provides useful clues about gene function, biochemical characterization of gene products remains essential to accurate annotation, particularly in cases where apparent functional redundancy is suggested based upon the presence of multiple homologous proteins. For example, a number of enzymes involved in vitamin B6 metabolic pathways remain uncharacterized, despite the important role this vitamin plays in *Mtb* metabolism. The term ‘vitamin B6’ collectively refers to pyridoxine 5′-phosphate (PNP) (**1**), pyridoxal 5′-phosphate (PLP) (**2**), pyridoxamine 5′-phosphate (PMP) (**3**), and their respective non-phosphorylated forms [2]. PLP biosynthesis is required for *Mtb* survival and virulence *in vivo* and it is a predicted cofactor to a number of enzymes essential to *Mtb* growth [3]. Therefore, vitamin B6 biosynthesis and salvage may be attractive target pathways for the development of novel compounds with anti-tuberculosis activity.

**Figure 1:**
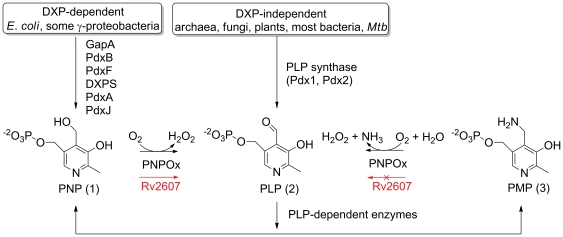
PLP formation catalyzed by PNPOx and associated vitamin B6 metabolic pathways. The role of Rv2607 is shown in red. In *E. coli*, PNPOx catalyzes the last step in the DXP-dependent PLP biosynthetic pathway [2]. Most organisms capable of PLP biosynthesis produce PLP via PLP synthase, a macromolecular complex consisting of Pdx1 and Pdx2 [2]. Organisms with genes that encode both PLP synthase and PNPOx likely use PNPOx to salavage PLP from PNP and PMP, which are produced by enzymes that use PLP as a cofactor.

In both prokaryotic and eukaryotic organisms, pyridoxine 5′-phosphate oxidase (PNPOx) is a flavin mononucleotide (FMN)-dependent enzyme encoded by the gene *pdxH* that produces PLP by oxidizing either PNP or PMP [4] (Figure 1). In *Escherichia coli*, PNPOx catalyzes the last step in the deoxyxyulose 5′-phosphate (DXP)-dependent biosynthetic pathway to PLP [2]. Most prokaryotes and plants biosynthesize PLP via an alternative route called the DXP-independent pathway [5] (Figure 1). The DXP-independent pathway includes PLP synthase, a macromolecular complex consisting of enzymes encoded by the genes *pdx1* and *pdx2* [2]. This complex is capable of carrying out *de novo* PLP biosynthesis directly. The *Mtb* genome encodes homologs of proteins involved in the DXP-independent PLP biosynthetic pathway, *pdx1* (Rv2606c) and *pdx2* (Rv2604c), but lacks the genes for enzymes in the DXP-dependent pathway [6]. It has been demonstrated that *Mtb* synthesizes PLP via the DXP-independent pathway using PLP synthase and that disruption of the *pdx1* gene generates a vitamin B6 auxotrophic *Mtb* mutant [3]. Organisms with genes that encode both PLP synthase and PNPOx probably only use the oxidase to salvage PLP after it participates as a cofactor in enzymatic reactions. It is unclear whether *Mtb* can acquire PLP or other B6 vitamers from the host [3].

The *Mtb* genome contains a gene for a putative PNPOx, Rv2607, annotated as *pdxH* (Cole 1998). Gene expression for *rv2607* (*pdxH*) has been detected in over 300 different experimental conditions in vitro ([7]; www.tbdb.org/expressionHistory.shtml?gn=Rv2607) as well as in macrophages [8]. Expression of this gene is upregulated by stresses such as exposure of *Mtb* to certain inhibitors of translation as well as by nitroimidazoles such as PA-824 [7]. Intriguingly, the *Mtb* genome encodes six gene products in addition to Rv2607 that are also annotated as PNPOx or “PNPOx-like” proteins and they have unknown function (Table 1). The PNPOx-like proteins (Rv1155, Rv2991, Rv2074, Rv1875, Rv3369, Rv0121c) appear to share global topology similarity with Rv2607, but display low overall sequence similarity (less than 25%). Rv2607 is the most highly conserved in this protein family across other mycobacterial species, including in the reduced genome of *M. leprae*, and it has the highest homology to the PNPOxs of *Streptomyces coelicolor* and *Saccharomyces cerevisiae*. It is, therefore, the most logical candidate for the canonical PNPOx of *Mtb*.

**Table 1:** Summary of NCBI gene annotations for PNPOx-like proteins in selected organisms that contain *pdx1* and *pdx2* and the role of PNPOx is considered to be for PLP salvage.

*M. tuberculosis*	*M. smegmatis*	PI	*M. leprae*	PI	*S. coelicolor*	PI
Rv2607 (PNPOx)	pdxH	(69%)	pdxH	(67%)	SCO4387	(44%)
Rv1155 (PNPOx-like)	MSMEG_5170	(80%)	ML1508	(88%)	SCO5312	(57%)
Rv2991 (PNPOx-like)	MSMEG_0048	(73%)	–		SCO1357	(38%)
Rv2074 (PNPOx-like)	MSMEG_3880	(85%)	–		SCO0838	(53%)
Rv0121c (PNPOx-like)	MSMEG_6526	(59%)	–		–	
Rv1875 (PNPOx-like)	MSMEG_6576	(35%)	–		–	
Rv3369 (PNPOx-like)	–		–		–	

Homologs were assigned using protein basic local alignment search tool (pBLAST) [26] for sequence alignments with E-values below 10^−6^. Percent identity (PI) is shown to the right of each gene annotation, which has been calculated by dividing the number of identical residues by the length of the alignment.

The crystal structure of Rv2607 has been solved to 2.5 Å resolution [9]. The proposed active site of Rv2607 is very similar to that of PNPOx homologs in other organisms. Residues known to be important for PLP binding in the *E. coli* and human PNPOx are nearly all conserved in Rv2607 (Figure S3) and a structural superimposition of the *E. coli* PNPOx and Rv2607 reveals a low RMSD of 0.8 Å for alpha carbons in the PLP binding region of the active site [9] (Figure S4). Although FMN could not be definitively placed in the *Mtb* PNPOx active site (electron density for the FMN cofactor is weak in the Rv2607 crystal structure), the residues known to interact with FMN in the *E. coli* and human PNPOx enzymes were found to be conserved in Rv2607 [9].

## Results and Discussion

To test the hypothesis that Rv2607 is a PNPOx, we heterologously overexpressed in BL21(DE3) *E. coli* cells and purified the amino-terminal 6x-histidine tag fusion protein. SDS-PAGE analysis showed that the protein was purified to >95% homogeneity and the yield of the purified protein was 140 mg per liter of cells, as determined using the Bradford test [10].

**Figure 2:**
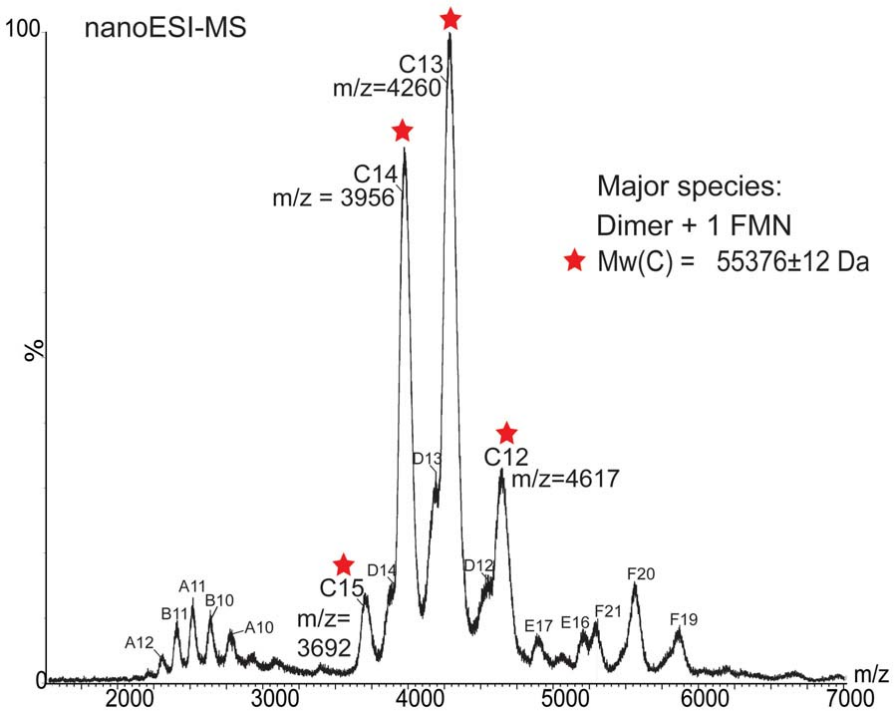
Nano-ESI mass spectrum of intact Rv2607 with co-purified FMN. The major species, *C*, has peaks with charges ranging from 15+ to 12+ in the 3500-4500 m/z range that correspond to a molecular weight of 55376±12. This experimental mass is consistent with the calculated mass for the complex of dimeric Rv2607 with one molecule of FMN bound (55442 Da), which is derived from the amino acid sequence of the 6x-histidine tagged Rv2607 (54986 Da; 27493 Da per protomer) and the molecular weight of FMN (456 Da). Monomeric tagged Rv2607 and truncated Rv2607 monomer (missing 10-11 residues), are present in solution and correspond to molecular weights of *A* (27450±52 Da) and *B* (26215±54 Da) respectively. A minor dimeric species comprised of full length and truncated monomer is represented as species *D*. Higher molecular weight oligomers (trimers *E* and tetramers *F* with molecular weights of 110.6 and 82.7 kDa, respectively) are electrospray-induced non-specific association of monomers and dimers.

The purified protein was bright yellow in color, suggesting the presence of a co-purified cofactor. The yellow cofactor bound tightly to Rv2607 as purified and was unambiguously identified as FMN (m/z 457.2 Da) by nanoflow electrospray ionization mass spectrometry (nESI-MS) after it was isolated from the heat-denatured protein (Figure S1). Intact Rv2607 analyzed by nESI-MS reveals it exists in a predominantly dimeric oligomerization state (Figure 2), which is consistent with other known PNPOxs [4]. This data, in combination with circular dichroism analysis, indicates that the freshly purified protein is functionally folded. Although two FMN binding sites exist at the homodimer interface of Rv2607, only one FMN molecule per dimer is present in the protein as purified (Figure 2). A 2:1 ratio of PNPOx protomer to FMN has been reported for other PNPOxs [11], [12], [13].

Two major protein bands were observed by SDS-PAGE, one corresponding to the Rv2607 monomer (∼27 kDa) and the other corresponding to a homodimer (∼54 kDa). The identity of the band corresponding to 54 kDa was confirmed as Rv2607 by MS analysis. In order to investigate the stability of the dimer, the charge state 14+ of Rv2607 was isolated in a tandem MS experiment (MS/MS) and submitted to collision-induced dissociation (CID). The MS/MS spectrum (Figure S2) shows that dissociation begins to occur at accelerating voltages of 150 V (trap) and 110 V (transfer). High accelerating voltages were required to induce dissociation of this very stable Rv2607 homodimer. Small populations of both monomers are visible at high and low m/z respectively and several additional peaks in the spectrum indicate that such high voltages also cause fragmentation of the protein, which has been previously reported for a different system [14]. This result is consistent with the crystal structure of Rv2607, which indicates that the two protomers are very tightly wrapped around each other and the dimer interface consists of about 20% of the total protomer surface [9].

**Figure 3:**
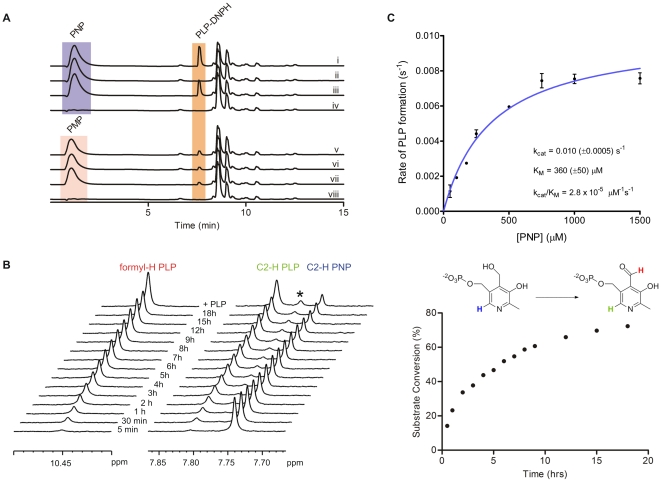
Reverse-phase HPLC, NMR, and spectrophotometric analysis of the PNPOx activity of Rv2607. (A) HPLC chromatograms (270 nm) of reaction mixtures containing PNP (i-iv) or PMP (v-viii), FMN, and Rv2607, and the associated control reactions. All reactions were carried out in 25 mM potassium phosphate buffer (pH 7.8), incubated at 25°C for 3 h, and quenched with DNPH (0.7 mM final concentration). Where present, the reaction components were at the following concentrations: 1 mM PNP or PMP, 10 µM FMN, and 10 µM Rv2607. Reaction mixtures contained: (i) PNP, FMN, and Rv2607, (ii) PNP and FMN (enzyme negative control), (iii) PNP and Rv2607 (no added FMN), (iv) FMN and Rv2607 (substrate negative control), (v) PMP, FMN, and Rv2607, (vi) PMP and FMN (enzyme negative control), (vii) PMP and Rv2607 (no added FMN), (viii) FMN and Rv2607 (substrate negative control). The peak to the right of PLP-DNPH is DNPH, which has a retention time of 8.6 min. (B) ^1^H NMR analysis of the conversion of PNP into PLP with time. The enzymatic reaction (1 mM PNP, 28 µM enzyme, 10% D_2_O in 25 mM potassium phosphate buffer, pH 7.8) was incubated for 18 hours at 298 K in the spectrometer. Left; stacked ^1^H NMR spectra recorded at various time points and after the addition of authentic PLP. The starred peak corresponds to PLP hydrate. Right; a plot of percent substrate conversion versus time. Substrate conversion was determined by comparing integrals of the C2-^1^H signals associated with PLP (7.80 ppm) to that of PNP (7.75 ppm). (C) Michaelis-Menten plot for Rv2607 with PNP as a substrate. The rate of PLP formation was monitored spectrophotometrically (λ_max_  =  388 nm, ε = 4900 cm^−1^M^−1^) for various concentrations of PNP. All solutions were made in 100 mM potassium phosphate buffer (pH 7.8).

Rv2607-mediated catalysis of PLP formation was examined by LC-MS (Figure 3A). To resolve the product, PLP, from the substrates, PNP and PMP, PLP was derivatized with 2,4-dinitrophenylhydrazine (DNPH) to form a PLP-DNP hydrazone. It has been previously reported that PLP readily forms a stable hydrazone with phenylhydrazine [15]. DNP hydrazones are not formed with either PNP or PMP because they lack a reactive aldehyde. A peak corresponding to the chemically-synthesized PLP-DNP hydrazone (retention time  =  7.60 min) was detected in the enzymatic reaction of Rv2607 with PNP. Electrospray ionization in the positive mode confirmed it to be the DNP hydrazone of PLP (m/z = 428). No such peak was observed in the enzyme and substrate (PNP) negative controls, however, a peak was observed in the absence of added FMN since the enzyme co-purified with FMN. The PLP-DNP hydrazone was not detected when PMP was tested as a substrate (Figure 3A). To eliminate the possibility that the PLP formation observed is due to the presence of contaminating indigenous *E.coli* PNPOx, we tested *E.coli* Bl21(DE3) cell lysates for PNPOx activity and showed that PLP can only be detected when Rv2607 is overexpressed. We also conducted PNPOx activity assays using an inactive PNPOx-like His_6x_-tagged protein expressed and purified in the same manner as was Rv2607 and did not detect PLP production. These data confirm that the observed PNP turnover is solely due to Rv2607. Further, *E. coli* PNPOx demonstrates PMP turnover [4], while Rv2607 does not.

Rv2607-catalyzed formation of PLP was also observed directly by ^1^H NMR spectroscopy in the presence of Rv2607 and PNP over time (Figure 3B). Increasing signals corresponding to the C2-^1^H and the aldehyde proton of PLP were observed with time with a concurrent decrease in signal from the C2-^1^H of PNP. Under these experimental conditions, there was 75% conversion of the substrate after 18 hours (Figure 3B). Spiking the reaction mixture with a synthetic standard of PLP confirmed that the observed chemical shifts in the reaction mixture correspond to PLP.

Rv2607-catalyzed PNP oxidation exhibited Michaelis-Menten kinetics with a k_cat_ and K_M_ of 0.01 s^−1^ and 360 µM respectively (Figure 3C). The reported k_cat_ and K_M_ for the *E. coli* enzyme and PNP are 0.2-0.8 s^−1^ and 2 µM, respectively [16], [17] and the k_cat_ and K_M_ for the human enzyme with PNP is 0.19 s^−1^ and 1.8 µM, respectively [18]. While both the *E. coli* and human PNPOx recognize PMP as a substrate, significant PLP formation was not seen when PMP was tested as a substrate for Rv2607. To our knowledge, Rv2607 is the first example of a PNPOx that does not oxidize PMP to PLP. The biological implication of the kinetic differences between Rv2607 and other PNPOxs is not yet known. It may be that Rv2607 exhibits a relatively low catalytic efficiency because it is not directly involved in the biosynthesis of PLP (as is the *E. coli* PNPOx) and unlike the human PNPOx, it is operating in a cellular environment which possesses the machinery for *de novo* PLP biosynthesis.

Although the *Mtb* protein encoded by Rv2607 was annotated as a member of the PNPOx family based on sequence alignment, biochemical data to support this classification had not been previously reported. PNPOx isolated from prokaryotic and eukaryotic sources (pig [11] and sheep [12] brain, rabbit liver [19], recombinant *E. coli* [16], recombinant human [18]) are reported to catalyze the oxidation of PNP as well as PMP to form PLP. We have found that Rv2607 catalyzes the oxidation of PNP, but not PMP, in the presence of FMN. Many organisms that are capable of *de novo* PLP biosynthesis possess homologs to PNPOx (Table 1), although there are exceptions, including *Bacillius subtillis.* The genomes of several microorganisms, including that of *Mtb* and other mycobacteria, include genes that encode for proteins structurally related to PNPOx and are annotated as ‘PNPOx-like proteins’. Very little is known about the function of these proteins. PNPOx-like proteins may have redundant PNPOx function or they may have evolved a new, as yet unknown function.

## Materials and Methods

### Overexpression and purification

The clone of Rv2607 was obtained as an amino-terminal 6x-histidine tag fusion construct with thrombin cleavage site in a modified pET28b vector from the laboratory of Dr. Thomas C. Terwilliger, Los Alamos National Laboratories, New Mexico, USA [9]. The construct also included a kanamycin resistance gene for selection. Rv2607 was transformed in *Escherichia coli* BL21-Gold(DE3) cells (Stratagene). An overnight culture of a single colony grown at 37°C with agitation was diluted 1∶100 into 500 mL Luria Broth (LB) supplemented with 40 µg/mL kanamycin. The culture was incubated at 37°C with shaking. Protein expression was induced when the culture reached an OD_600nm_ of 0.6 with the addition of isopropyl thiogalactopyranoside (IPTG) to a final concentration of 1 mM. The culture was further incubated at 20°C for 20 h with agitation. Cells were harvested by centrifugation (14000x g, 15 min). The bright yellow cell pellet was resuspended in 20 mM Tris-HCl, 100 mM NaCl, pH 8.0. The cells underwent one freeze-thaw cycle (−80°C) and were further lysed by sonication (Misonix S-4000, amplitude 60%, 1 s on, 1 s off for 30 s, repeated 3 times). The cell lysate was centrifuged (48,000 x g, 30 min) and the clarified yellow supernatant was applied to a Ni-NTA affinity column (5 mL) pre-equilibrated with buffer A (20 mM Tris-HCl, 100 mM NaCl, 20 mM imidazole, pH 8.0). The column was washed with buffer A (150 mL) until the flow through no longer contained any protein, as determined by a Bradford test [10]. The protein was eluted with buffer B (20 mM Tris-HCl, 500 mM NaCl, 300 mM imidazole, pH 8.0). The eluted protein was buffer exchanged into phosphate buffered saline (PBS) (pH 7.6) containing 10% glycerol using Vivaspin 20 10 kDa molecular weight cut off centrifugal filters. SDS-PAGE analysis showed that the protein was purified to >95% homogeneity and the yield of the purified protein was determined to be 140 mg per liter of cells with the Bradford assay.

### Synthesis of PNP

PNP was synthesized based on a published procedure [20] with some modifications. Under argon atmosphere, freshly prepared 0.5 M NaOH (3.2 mL) was added to PLP (200 mg, 0.8 mmol). The solution was deoxygenated by bubbling with argon for 5 min at room temperature, after which sodium borohydride (12 mg, 0.32 mmol) was added portionwise with stirring. The reaction was stirred until complete as judged by TLC (SiO_2_, 80∶20 methanol: ethyl acetate v:v). The reaction was quenched with acetone (120 µL, 1.6 mmol) and allowed to stir at room temperature for 30 min, after which NaOH (147 mg, 3.7 mmol) was added. After stirring for another 15 min at room temperature, the reaction mixture was loaded directly onto a column containing 5 mL Amberlite IRA-743 (5 mL in a 1.0×11.0 cm column) to remove borate salts. The resin had been conditioned by washing with absolute ethanol (25 mL), water (3×25 mL), 1 M HCl (25 mL), water (25 mL), 1 M NaOH (25 mL), and finally equilibrated with water (50 mL). The flow through was collected and the resin was washed with enough water (5-7 mL) so that PNP could no longer be detected spectrophotometrically (λ_max_  =  325 nm at pH 7.5). The flow-through and wash were pooled and this solution was further purified with weakly acidic cation exchange resin Amberlite IRC-76 (10 mL in a 1.30×12.5 cm column). The resin had been conditioned with 1 M NaOH (50 mL), water (50 mL), 1 M HCl (50 mL), and finally equilibrated with water (100 mL). The flow through was collected and pooled with two elution steps: 0.5 M NaOH (5 mL) and water (10 mL). The resin was washed with water (10 ml) until PNP could not be detected spectrophotometrically. The eluted product was pooled, lyophilized, and resuspended in 100 mM potassium phosphate buffer, pH 7.8. ^1^H NMR (D_2_O, 400 MHz): δ 7.77 (1H, s), 4.88 (2H, d, *J = 6.9*), 4.77 (2H, s), 2.41 (3H, s).

### Formation of dinitrophenylhydrazone of PLP

Dinitrophenylhydrazine (DNPH) reactivity with PLP, PNP, and PMP was tested. To 1 mM solutions of PLP (100 µL), PNP (100 µL), and PMP (100 µL) in 25 mM potassium phosphate buffer (pH 7.8) a saturated solution of DNPH in 3 M HCl (10 µL) was added to a final concentration of 0.7 mM. As a negative control, 3 M HCl (10 µL) was substituted for DNPH. The PLP, PNP and PMP negative control reactions were carried out by substituting the substrate with 100 µL of 25 mM potassium phosphate buffer (pH 7.8). All reactions were carried out at 25°C for 3 h.

### Assay for Rv2607-dependent PLP formation

Reaction mixtures (150 µL each) containing 1 mM PNP or 1 mM PMP (Figure 2), 10 µM FMN, and 10 µM freshly purified Rv2607 in 25 mM potassium phosphate buffer were incubated at 25°C for 3 h. Enzyme, cofactor, and substrate negative control reactions were treated in a similar manner. After 3 h incubation at 25°C, the reaction was quenched with addition of 10 µl of DNPH in 3 M HCl to a final concentration of 0.7 mM. The reaction mixture was filtered with 10 kDa MW cutoff centrifugation filters and 10 µl of the reaction was analyzed by LC-MS.

### LC-MS analysis

Analysis of the enzymatic reactions and controls were performed on Agilent 1100 LC-MS instrument using Luna (3 µm) C18 column (50×2 mm, Phenomenex). Solution A contained water and 0.1% formic acid. Solution B contained acetonitrile and 0.1% formic acid. The column was equilibrated with 95% solution A for 1 min. The following gradient was used: 5% to 95% solution B from 1 to 11 min; 95% solution B from 11 to 15 min; 95% to 5% solution B from 15 to 17 min; 5% solution B from 17 to 21.5 min.

### NMR time-course

The enzymatic reaction was monitored by ^1^H NMR spectroscopy using a Bruker Avance 700 MHz spectrometer with a TXI cryoprobe. The reaction mixture contained 1 mM PNP, 10% deuterium oxide, and 20 μM 2,2,3,3-D4-3-(trimethylsilyl)propionic acid (TSP) as an internal standard in 25 mM potassium phosphate buffer (pH 7.6). The reaction was initiated with the addition of freshly purified enzyme to a final concentration of 28 µM or an equivalent volume of buffer for the control. The enzymatic reaction and enzyme negative control (200 µL) were added to 3 mm capillary tubes (Bruker), which were maintained at constant temperature (25°C). The spectrometer conditions were optimized with the enzyme negative control sample. The sample was locked, shimmed, and the pulses were calibrated for water suppression using excitation sculpting with gradients [21]. ^1^H NMR spectra were collected at selected time points for 18 hours (Figure 3). Each experiment consisted of 4 dummy scans followed by 8 collected scans, resulting in 63 s of acquisition time per data point. Chemical shifts are reported in ppm relative to TSP (δ = 0 ppm). The spectra were processed and analyzed using TopSpin™ 1.8 (Bruker, Billerica, Massachusetts, USA). Substrate conversion was determined by comparing integrals of the C2-^1^H signals associated with PLP (7.80 ppm) to that of PNP (7.75 ppm) (Figure 3B). To confirm the identity of the product formed, the reaction mixture was enriched with authentic PLP after 18 hours.

### Steady state kinetic parameters

The rate of product formation was monitored spectrophotometrically with a Varian Cary 400 UV/Vis spectrophotometer. The product, PLP, has a λ_max_ at 388 nm and an extinction coefficient of 4900 cm^−1^M^−1^ [22]. The rate of PLP production was measured for various concentrations of PNP: 50 µM, 100 µM, 175 µM, 250 µM, 500 µM, 750 µM, 1000 µM, 1500 µM. Freshly purified enzyme was added to each reaction mixture and no exogenous FMN was added. All solutions were made in 100 mM potassium phosphate buffer (pH 7.8). The K_M_ and k_cat_ for Rv2607 were determined by fitting the initial rate (less than 10% conversion) of product formation as a function of substrate concentration to the Michaelis-Menten equation by non-linear regression using GraphPad Prism 5 (GraphPad Software, Inc., La Jolla, California, USA) (Figure S1).

### Mass spectrometry

Nanoflow electrospray ionization mass spectra (nESI-MS) were recorded on a Synapt HDMS system optimized for the transmission of noncovalent complexes [23]. Typically, 2.5 µL solutions containing Rv2607 in 100 mM ammonium acetate were electrosprayed from gold-coated glass capillaries [24]. To preserve the non-covalent interactions in the Rv2607 dimer in complex with FMN, the MS parameters used for the Synapt were: capillary voltage, 1.7 kV; sample cone, 80–100 V; trap and transfer collision energy, 15V and 12 V respectively; backing pressure 3.8 mbar; trap and IMS pressure 5e^−2^ and 5e^−1^ mbar, respectively; time-of-flight analyzer pressure 1.16e^−6^ mbar. For the CID experiment, the “trap and transfer collision energies” were 150 V and 110 V respectively to effect dissociation. Extracted cofactor from Rv2607 and authentic FMN were analyzed with a Q Star Elite Mass Spectrometer (Applied Biosystems/MDS Sciex) by electrospray ionization positive ion mode with a gold-plated nanospray tip (Figure S1). To extract the bound cofactor from Rv2607, a concentrated solution of purified Rv2607 (270 µM) in 25 mM potassium phosphate buffer (pH 7.8) was heat-denatured for 5 min at 100°C. To the denatured protein was added 300 µL 25 mM potassium phosphate buffer (pH 7.8) and the mixture was filtered using a 10 K molecular weight cutoff Vivaspin 500 centrifugation filter (15 min at 17,900 *x g)*. The flow through was desalted with C18 Zip Tip® pipette tips previously washed with acetonitrile and equilibrated with 0.1% trifluoroacetic acid (TFA) in ultrapure water. The analyte was eluted in a solution of acetonitrile and 0.05% TFA. The FMN standard sample was acidified in 0.1% TFA to a final concentration of 500 µM. All spectra were calibrated internally using a solution of cesium iodide (100 mg/mL). Data were processed with MassLynx 4.0 software (Waters/Micromass).

## Supporting Information

Figure S1 Identification of cofactor bound to Rv2607 by electrospray ionization mass spectrometry. Cofactor was extracted from Rv2607 by heat denaturation. FMN has a molecular weight of 456.2 Da.
(TIF)

Figure S2 MS/MS spectra of Rv2607 homodimer. The isolated precursor ion, charge state 14+ of the dimeric Rv2607 complex was submitted to CID with accelerating voltages 150 V (Trap) and 110 V (Transfer). Low populations of charge-state series A and B corresponding to monomers with measured masses 27362 Da (monomer, high m/z region) and 27353 Da (low m/z region) begins to occur at these high voltages. High accelerating voltages also caused fragmentation of the protein, indicated by a few unassigned peaks at the low and high m/z region.
(TIF)

Figure S3 Protein sequence alignment of Rv2607 and *E. coli* PNPOx. Residues highlighted in red are highly conserved motifs in PNPOxs [25]. The arrow points to a key difference in the *E. coli* PNPOx and Rv2607 sequence that results in greater solvent-exposure of the active site in Rv2607.
(TIF)

Figure S4 Structural and mechanistic analysis of Rv2607-catlyzed oxidation of PNP to PLP. (A) Superimposition of the active site residues of Rv2607 (yellow, PDB ID: 2A2J) and *E. coli* PNPOx (grey, PDB ID: 1G77). Labeled residues correspond to Rv2607. PLP and FMN are present in the *E. coli* PNPOx structure only. (B) Proposed mechanism for the oxidation of PNP. A hydride is transferred from PNP to FMN and oxidized FMN is regenerated with molecular oxygen.
(TIF)
