# Supplementary figures and images for: Rv2607 from Mycobacterium tuberculosis Is a Pyridoxine 5′-Phosphate Oxidase with Unusual Substrate Specificity
Source: PLoS One. 2011 Nov 14;6(11):e27643. doi: 10.1371/journal.pone.0027643 (PMC3215729; doi:10.1371/journal.pone.0027643)

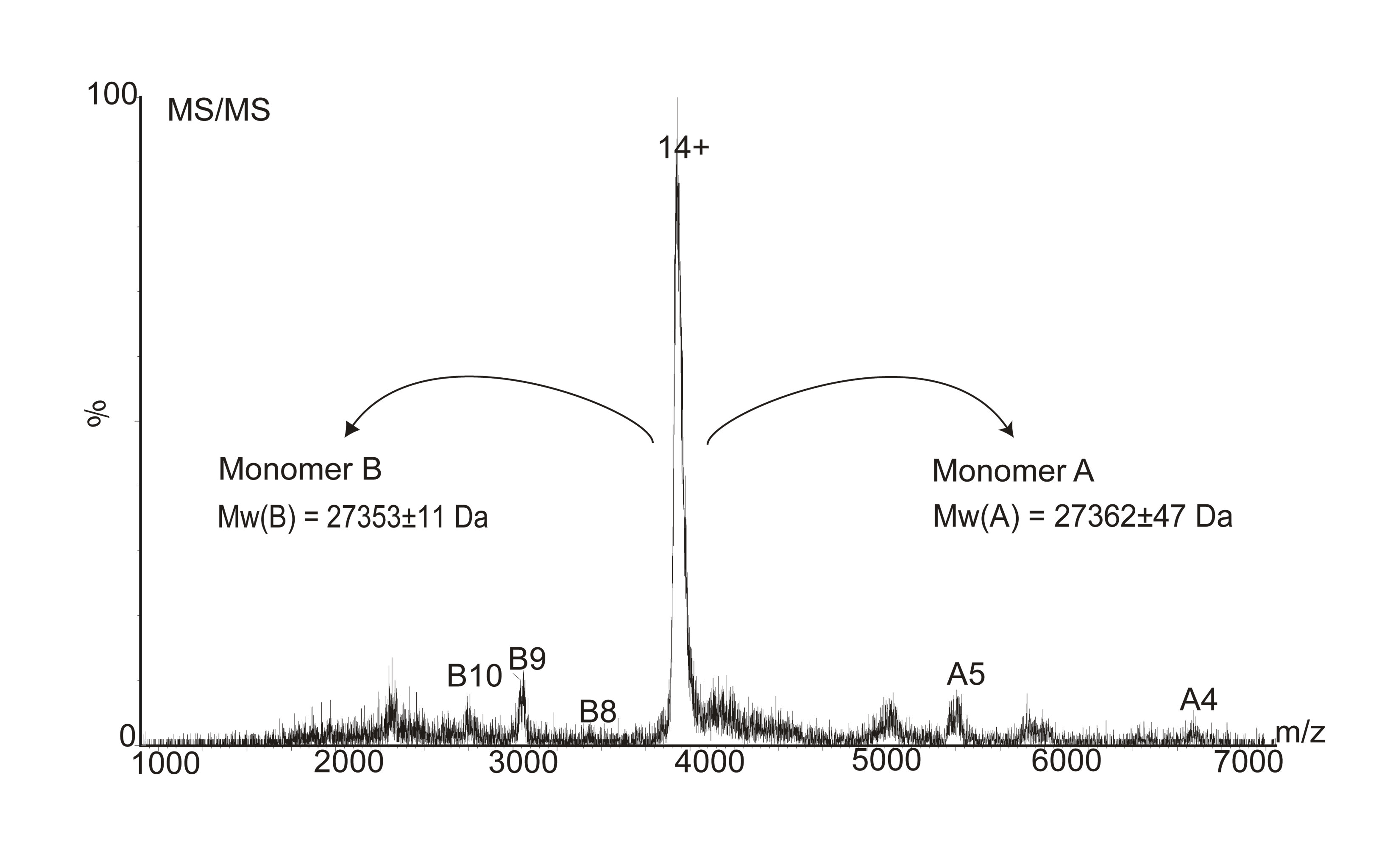

Supplement: Figure S2 — MS/MS spectra of Rv2607 homodimer. The isolated precursor ion, charge state 14+ of the dimeric Rv2607 complex was submitted to CID with accelerating voltages 150 V (Trap) and 110 V (Transfer). Low populations of charge-state series A and B corresponding to monomers with measured masses 27362 Da (monomer, high m/z region) and 27353 Da (low m/z region) begins to occur at these high voltages. High accelerating voltages also caused fragmentation of the protein, indicated by a few unassigned peaks at the low and high m/z region. (TIF) [file pone.0027643.s002.tif]

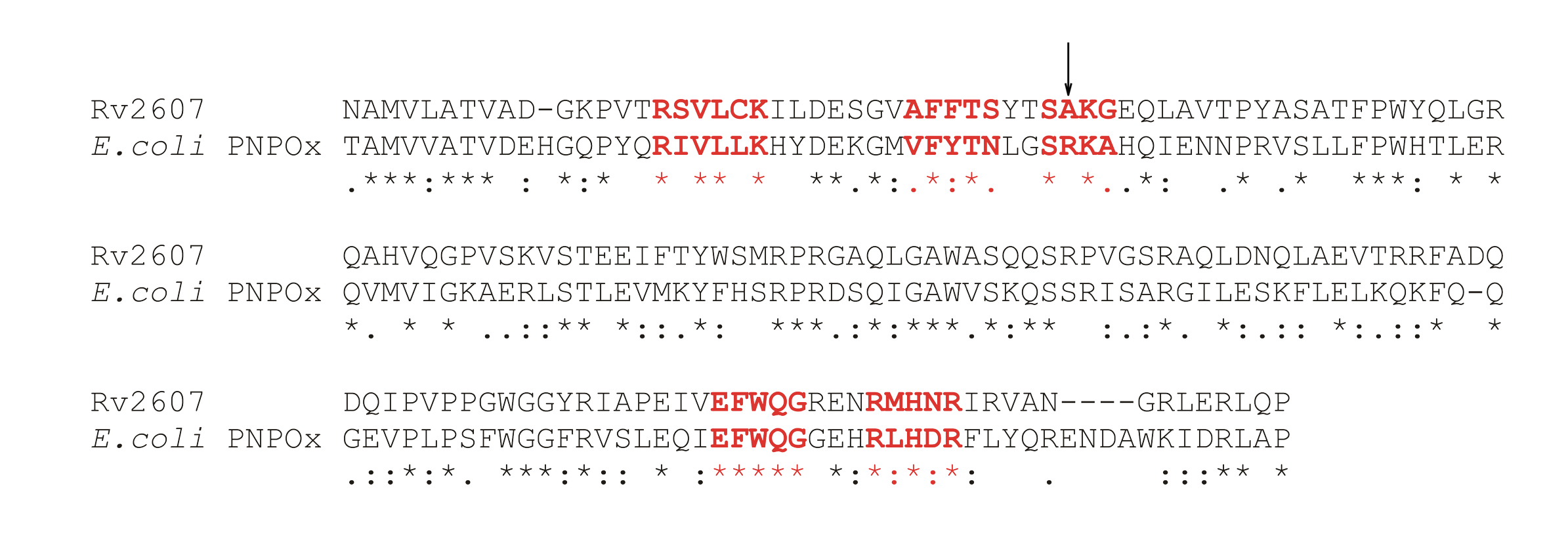

Supplement: Figure S3 — Protein sequence alignment of Rv2607 and E. coli PNPOx. Residues highlighted in red are highly conserved motifs in PNPOxs [25]. The arrow points to a key difference in the E. coli PNPOx and Rv2607 sequence that results in greater solvent-exposure of the active site in Rv2607. (TIF) [file pone.0027643.s003.tif]

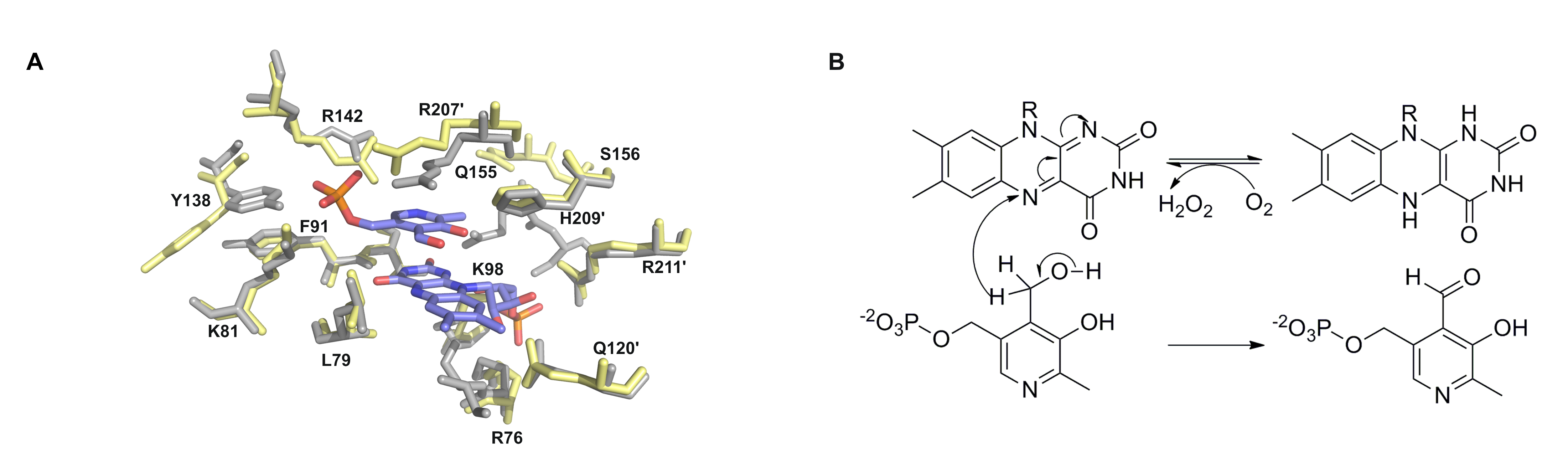

Supplement: Figure S4 — Structural and mechanistic analysis of Rv2607-catlyzed oxidation of PNP to PLP. (A) Superimposition of the active site residues of Rv2607 (yellow, PDB ID: 2A2J) and E. coli PNPOx (grey, PDB ID: 1G77). Labeled residues correspond to Rv2607. PLP and FMN are present in the E. coli PNPOx structure only. (B) Proposed mechanism for the oxidation of PNP. A hydride is transferred from PNP to FMN and oxidized FMN is regenerated with molecular oxygen. (TIF) [file pone.0027643.s004.tif]
